# Atypical Presentation of a Urachal Carcinoma as an Enterocutaneous Fistula

**DOI:** 10.7759/cureus.23697

**Published:** 2022-03-31

**Authors:** Ana Rita Martins, Joana Frazão, Sara Nogueira, António Godinho

**Affiliations:** 1 General Surgery, Hospital São Francisco Xavier, Lisboa, PRT; 2 General Surgery, Hospital Professor Doutor Fernando Fonseca, Lisboa, PRT; 3 General and Colorectal Surgery, Clinical Institute of Digestive and Metabolic Diseases, Hospital Clínic, Barcelona, ESP

**Keywords:** surgery, enterocutaneous fistula, atypical presentation, aggressive tumor, urachal carcinoma

## Abstract

Urachal carcinoma is a rare and aggressive tumor, involving the urachus and the bladder. Symptoms of urachal carcinoma usually appear at later stages of the disease; therefore, these tumors are diagnosed in advanced stages, providing limited options for curative treatment.

We report the clinical case of a 60-year-old man with a urachal carcinoma which presented as a mass of the abdominal wall invading the transverse colon, creating an enterocutaneous fistula. The patient underwent an en-bloc resection of the mass, segmentary resection of the transverse colon, and partial cystectomy.

## Introduction

Urachal carcinoma is a rare and highly aggressive tumor, involving the urachus and the bladder. Because it is an extremely rare cancer, it often goes misdiagnosed, making it difficult to determine its true incidence in the general population. It represents less than 1% of all bladder cancers [1,2] and 0.01% of all cancers [3].

Symptoms usually appear late in the disease and the diagnosis is often made at an advanced stage [4,5]. The treatment consists of partial cystectomy as well as resection of the median umbilical ligament and umbilicus [3,6].

We present an atypical presentation of urachal carcinoma and a review of the most relevant literature.

## Case presentation

A 60-year-old male, with a history of known alcohol abuse and smoking, was admitted to the emergency department due to an abdominal mass and unexplained weight loss (20% in four months), without gastrointestinal or urinary symptoms.

On physical examination, a large round mass was detected and located just below the umbilicus with a size of 10 cm, showing hard consistency and inflammatory signs. A high C-reactive protein (CRP) level combined with a high white blood count (WBC) confirmed the suspicion of an inflammatory process. The serum levels of carcinoembryonic antigen (CEA) were also elevated.

**Figure 1:**
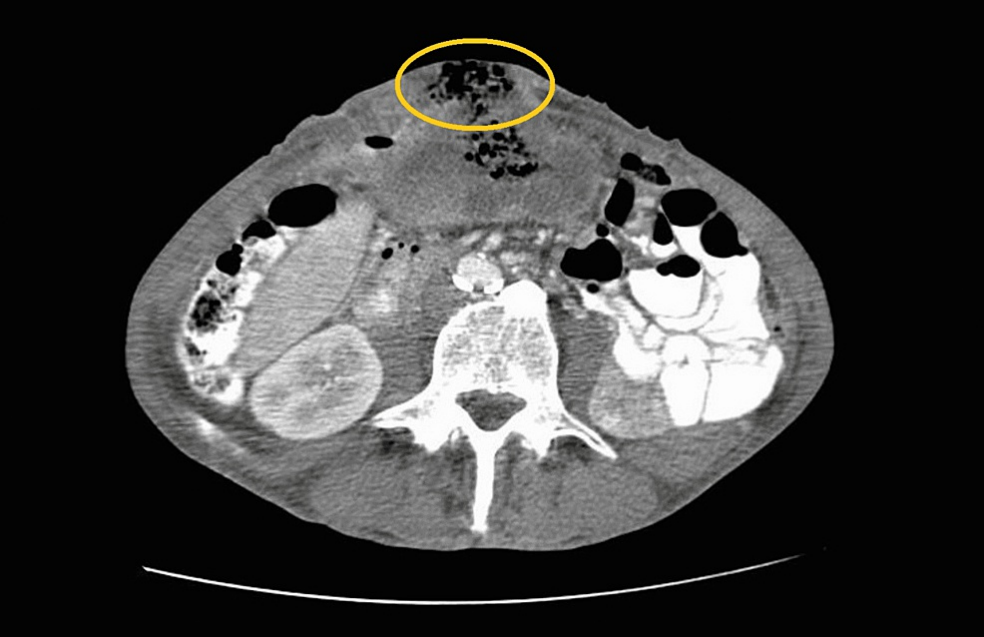
Coronal sections of abdominal CT images indicate a heterogeneous mass, presenting as enterocutaneous fistula (yellow circle).

**Figure 2:**
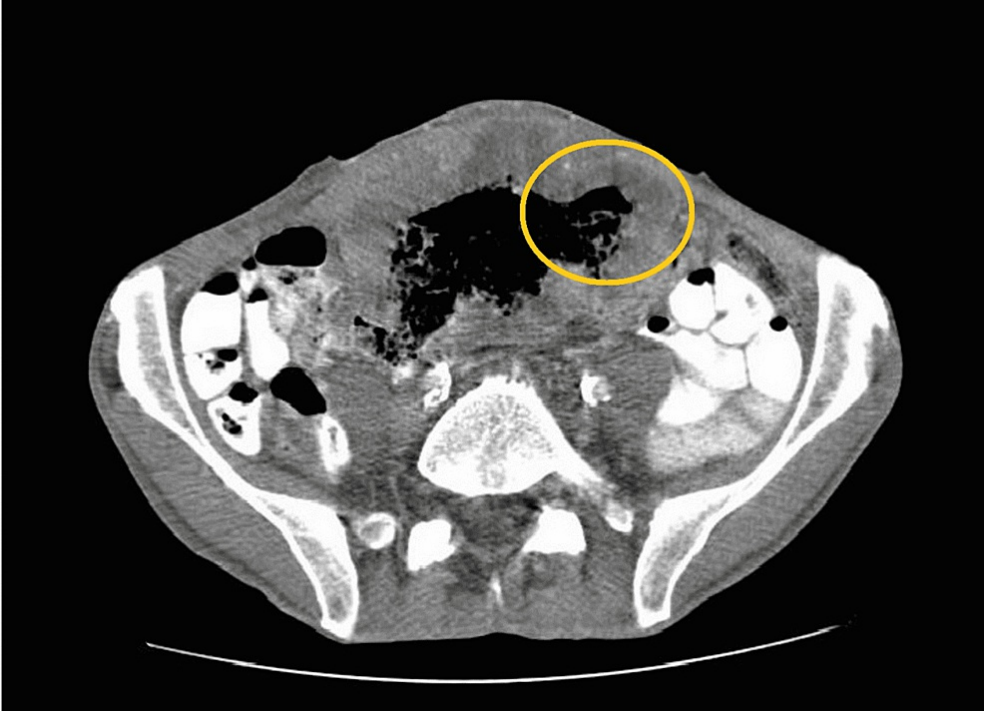
Coronal sections of abdominal CT images revealed a mass of the abdominal wall invading the colon (yellow circle).

The abdominal CT scan revealed a 14x6x10 cm mass, extending from the umbilicus to the vesical cupula, heterogeneous, and with microcalcifications, causing deviation of intra-abdominal organs, with doubtful origin: urachus versus colonic (Figures 1-2). The CT scan of the preoperative staging thorax was normal.

The patient was admitted for further study and antibiotic treatment and was discharged after nine days with a scheduled colonoscopy and cystoscopy a week later.

The colonoscopy was incomplete due to a fixation of the sigmoid colon that did not allow the progression of the endoscope. Around 24 hours after the exam, the patient presented to the ED passing feces through a newly formed orifice in the middle of the mass. After this, the patient was submitted to an emergent exploratory laparotomy.

**Figure 3:**
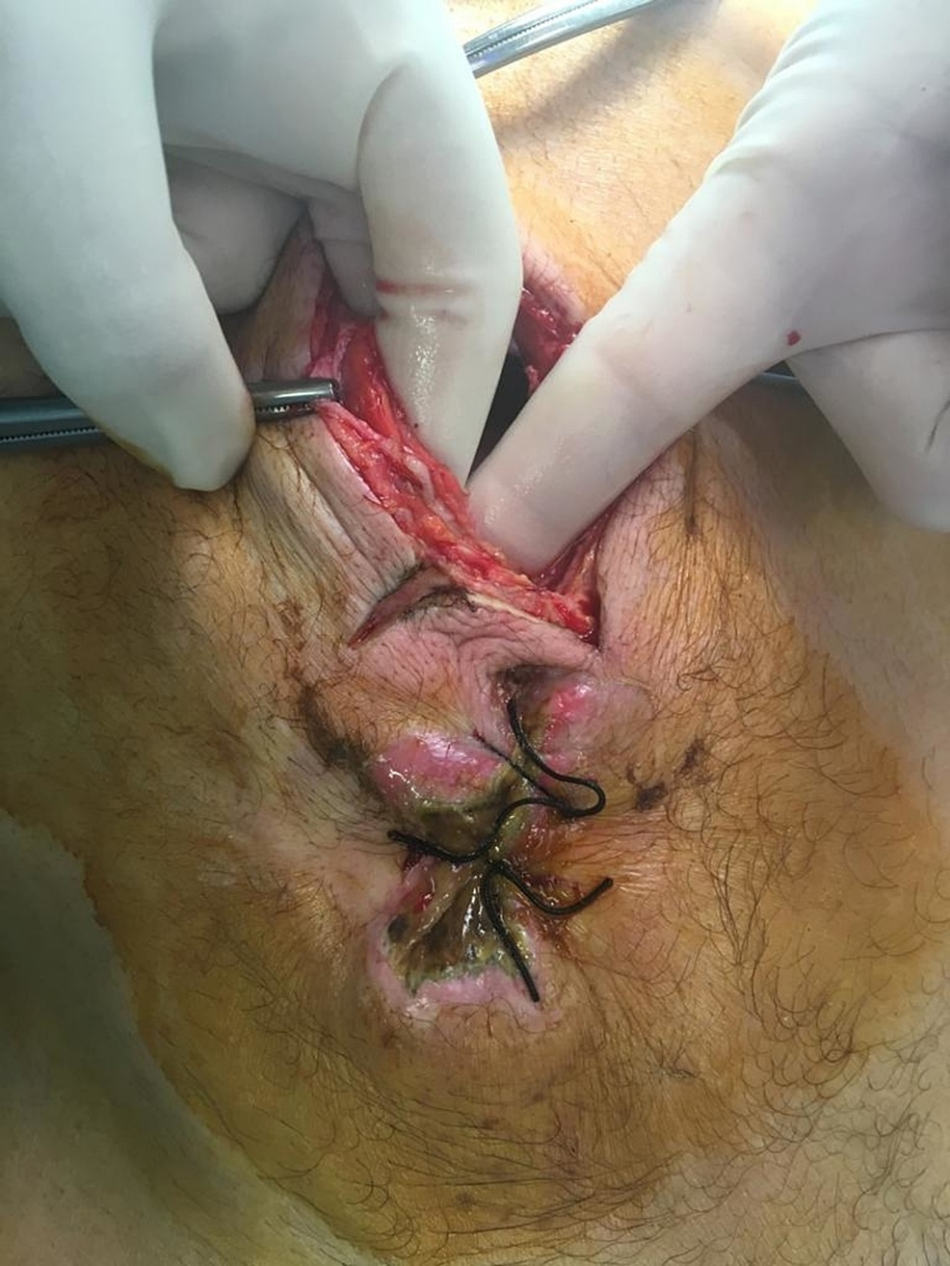
Atypical presentation as an enterocutaneous fistula.

**Figure 4:**
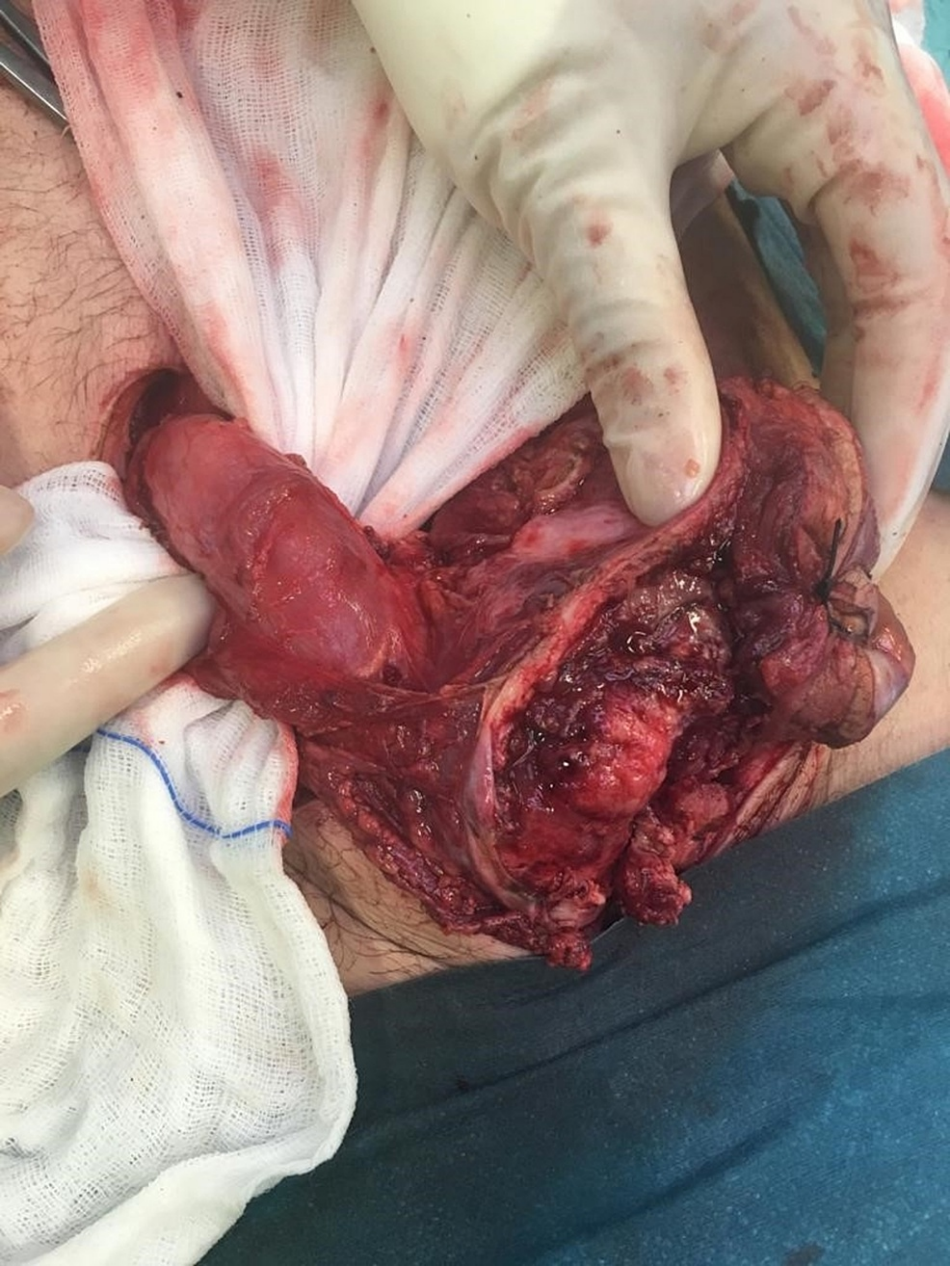
Mass of the abdominal wall invading the transverse colon.

**Figure 5:**
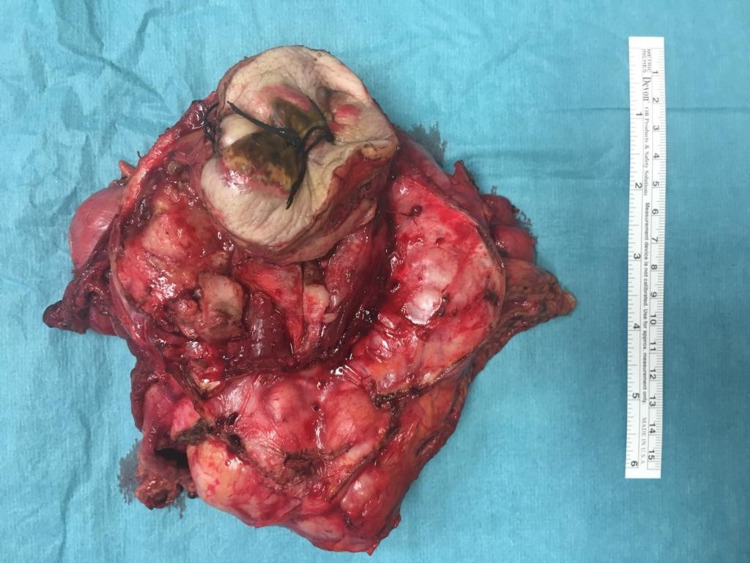
En-bloc resection of the mass, segmentary resection of the transverse colon and partial cystectomy

Intra-operative findings revealed a mass of the abdominal wall invading the transverse colon in contact with the upper part of the bladder (Figures 3-4). The surgical team performed an en-bloc resection of the mass and involved structures (segmentary resection of the transverse colon, partial cystectomy, and partial resection of the abdominal wall) (Figure 5). It was decided to leave a terminal colostomy of the transverse colon and delay reconstruction. 

The pathological analysis revealed an adenocarcinoma of the urachus, poorly differentiated (G3), infiltrating the resected colon and the bladder, with positive microscopic margin at the anterior and bladder margins (R1), negative lymph node, and no perineural or angioinvasion.

Two months after surgery, a CT scan showed a local recurrence in the bladder and diffuse bowel wall thickening. This case was discussed in a cancer multidisciplinary team meeting that proposed postoperative chemotherapy using the FOLFOX regimen.

The patient died one year later with peritoneal carcinomatosis and pulmonary metastasis.

## Discussion

The urachus is the structure that connects the bladder to the umbilicus of the fetus. Its lumen gradually disappears during embryonic development and after birth, it remains only as a fibrous cord, known as the median umbilical ligament [1,3,6]. In adults, the urachal remnants can still be found, commonly at the bladder dome [2,4,7,8]. The first description of urachal adenocarcinoma was made in 1863 [3,5,9]. Since then, more case reports and series have been described in the literature.

The most common histologic subtype is the adenocarcinoma with enteric features, with or without mucin production [1]. Approximately 70% of urachal adenocarcinomas are mucin-producing tumors [4,5,10].

Urachal tumors are more common among males and diagnosis is usually made between 50 and 60 years of age [10-12]. Clinical presentation is not specific, however, the most common feature is the presence of urinary symptoms. Around 52% to 82% of patients present with hematuria, which occurs due to bladder invasion. Therefore, hematuria is a strong predictor of urachal malignancy [3,8,10]. Less frequent symptoms may include a palpable suprapubic mass, mucus in the urine (mucinuria), pyuria, and recurrent urinary tract infections [7,10]. This case report describes an atypical and extremely rare presentation of a urachal carcinoma, such as an abdominal wall mass invading the transverse colon, creating an enterocutaneous fistula. This case fits in existing literature as it reinforces further the knowledge around this rare disease.

These tumors often grow for a long period of time prior to diagnosis, during months or even years before any symptoms appear [1]. Consequently, it is not easily detected in the early stages [5]. The criteria for the diagnosis of urachal carcinoma were defined by Sheldon et al. and Mostofi et al. and revised by Gopalan et al. These included a dome tumor or elsewhere in the midline of the bladder, absence of cystitis cystic and cystitis glandularis, a sharp demarcation between the tumor and normal surface epithelium, enteric-type histology, and absence of a primary neoplasm elsewhere [3,6-7,13-15]. These criteria were further adapted and published by the World Health Organization [9]. MD Anderson Cancer Centre simplified the diagnosis of urachal carcinoma to any enteric-type adenocarcinoma with sharp demarcation between the tumor and the epithelium in the bladder [16].

The diagnosis can be suspected based on a CT scan, which is useful to evaluate local invasion, nodal status and to detect the presence of distant metastases [10,13]. CT scans can help in distinguishing a benign mass from a malignant mass, but the definitive diagnosis is confirmed usually by cystoscopy, endoscopic biopsy, or at the time of surgery [1].

Different staging systems for urachal carcinoma have been proposed, such as the Sheldon, Mayo, and Ontario staging systems [2-3,7-8,11,16]. However, their significance still needs validation in a larger series [9]. The applicability of the TNM staging system is limited for urachal carcinoma [13-14]. A large proportion of patients present at stage III or higher, representing local invasion [7,11].

There are no standardized treatment protocols for these patients [5]. Reports suggest that surgery remains the main therapeutic option for localized disease, while chemotherapy schemes have been used in metastatic urachal cancer [12].

The surgical treatment is partial cystectomy with en-bloc resection of the median umbilical ligament up to the umbilicus [3,6-7,13]. This is the treatment of choice, required to appropriately control the tumor [17,18]. Partial cystectomy offers similar outcomes to complete cystectomy, providing a higher quality of life [9-11,16]. However, to achieve negative margins, a complete cystectomy might be performed [6]. Furthermore, some studies suggest that a failure to resect the umbilicus has been associated with a higher risk of relapse and a poorer survival rate [7,11,13]. Other prognostic factors in urachal carcinoma have been described, such as pathologic stage, positive surgical margins, positive lymph nodes, and involvement of the peritoneal surface at the time of surgery [1,6,8-9,13]. Lymph node dissection might be recommended, but there is still doubt regarding the benefit of lymphadenectomy [8,10-11,16,18].

Different chemotherapy regimens have been used as adjuvant therapy and in metastatic urachal cancer. It can include cisplatin and 5-fluorouracil-based combination therapies [4,10]. However, there is no current standard chemotherapy regimen for these patients and it´s yet unclear whether chemotherapy or radiation brings any benefit [1,3,5-6,12].

Biological treatment, immunotherapy, and targeted therapies are being studied as potential treatments. More research is necessary to determine what factors play a role in the development of urachal cancer [16,19].

The local recurrence of these tumors is high, mainly in the first two years after surgery, with rates of 38% in the pelvis and 34% in the bladder. The most common metastatic sites are the lung, bones, liver, and peritoneum [6,10,12,18].

Urachal carcinoma has a very poor prognosis, due to a high frequency of advanced disease at the time of diagnosis, local recurrence, and distant metastasis [1,4,8]. In the literature, the global five-year survival rate ranges between 43% and 61% [3,10,13,17].

## Conclusions

Urachal carcinoma is an extremely rare and aggressive malignancy, described in the literature only as single case reports or small case series. We present an atypical presentation of urachal carcinoma as a mass of the abdominal wall invading the transverse colon, creating an enterocutaneous fistula. This case can bring more information about this rare disease and its possible presentations.

Early recognition and diagnosis of this entity are very difficult, because of the silent nature of early lesions. An appropriate surgery with extensive tumor resection can provide the best chance and remains the mainstay of its therapy. However, many patients show advanced disease at diagnosis, leading to a very poor prognosis. Existing chemotherapy regimens and biological treatments can be used, but further studies are necessary to determine the role and effectiveness of these therapies for urachal cancer. Perhaps new immunotherapies will expand existing boundaries to improve results. More studies and prospective trials are necessary to clarify this rare disease.
